# Species of *Pseudorhabdosynochus* (Monogenea, Diplectanidae) from Groupers (*Mycteroperca* spp., Epinephelidae) in the Mediterranean and Eastern Atlantic Ocean, with Special Reference to the ‘Beverleyburtonae Group’ and Description of Two New Species

**DOI:** 10.1371/journal.pone.0159886

**Published:** 2016-08-17

**Authors:** Amira Chaabane, Lassad Neifar, Delphine Gey, Jean-Lou Justine

**Affiliations:** 1 Laboratoire de Biodiversité et Écosystèmes Aquatiques, Faculté des Sciences de Sfax, Université de Sfax, Sfax, Tunisia; 2 UMS 2700 Service de Systématique moléculaire, Muséum National d'Histoire Naturelle, Sorbonne Universités, Paris, France; 3 ISYEB, Institut Systématique, Évolution, Biodiversité, UMR7205 (CNRS, EPHE, MNHN, UPMC), Muséum National d’Histoire Naturelle, Sorbonne Universités, Paris, France; Institut national de la santé et de la recherche médicale - Institut Cochin, FRANCE

## Abstract

*Pseudorhabdosynochus* Yamaguti, 1958 is a species-rich diplectanid genus, mainly restricted to the gills of groupers (Epinephelidae) and especially abundant in warm seas. Species from the Mediterranean are not fully documented. Two new and two previously known species from the gills of *Mycteroperca* spp. (*M*. *costae*, *M*. *rubra*, and *M*. *marginata*) in the Mediterranean and Eastern Atlantic Ocean are described here from new material and slides kept in collections. Identifications of newly collected fish were ascertained by barcoding of cytochrome c oxidase subunit I (COI) sequences. *Pseudorhabdosynochus beverleyburtonae* (Oliver, 1984) Kritsky & Beverley-Burton, 1986 and *P*. *sosia* Neifar & Euzet 2007 are redescribed from type-specimens and new specimens collected off Tunisia and Libya from *M*. *marginata* and *M*. *costae*, respectively. *Pseudorhabdosynochus oliveri* n. sp., from *M*. *marginata* (type-host) off the Mediterranean coast of France (type-locality), is described from specimens found among voucher specimens of *P*. *beverleyburtonae* deposited by Guy Oliver in the collection of the Muséum National d’Histoire Naturelle, Paris. *Pseudorhabdosynochus oliveri* is distinguished by the shape of its sclerotised vagina; it was not found in the other localities investigated. *Pseudorhabdosynochus hayet* n. sp. is described from *M*. *rubra* (type host) off Senegal (type-locality) and Tunisia. *Pseudorhabdosynochus hayet* is morphologically similar to *P*. *sosia* (type-host: *M*. *costae*) but was distinguished by differences in measurements of the vagina and male copulatory organ, different host, and divergent COI sequences. The four species (*P*. *beverleyburtonae*, *P*. *sosia*, *P*. *oliveri*, and *P*. *hayet*) share common characteristics such as squamodiscs with 2 innermost circular rows of rodlets and a similar general structure of the sclerotised vagina; we propose to group them into a ‘beverleyburtonae group’ within *Pseudorhabdosynochus*.

## Introduction

*Pseudorhabdosynochus* Yamaguti, 1958 is a species-rich diplectanid genus [1–4]; its members are mainly restricted to the gills of groupers (Epinephelidae), with a few exceptions, and, since most groupers inhabit warm seas, they are especially numerous in tropical seas [5]. Species from the Mediterranean are not fully documented, although eleven nominal species have been listed [6, 7].

*Pseudorhabdosynochus beverleyburtonae* (Oliver, 1984) Kritsky & Beverley-Burton, 1986 is a gill parasite of the dusky grouper *Mycteroperca marginata* in the Mediterranean Sea and both sides of the Atlantic [2, 8–11]. We found that three species of *Pseudorhabdosynochus* from groupers assigned to *Mycteroperca* Gill in the Mediterranean Sea and Eastern Atlantic Ocean were morphologically very close to *P*. *beverleyburtonae*. Among these species, one, namely *P*. *sosia* Neifar & Euzet, 2007, was already known, and two are new and are described in this paper. Based on the sclerotised vagina, the primary character for species diagnosis within *Pseudorhabdosynochus*, we propose the ‘beverleyburtonae group’ to accommodate them. Monogenean COI sequences were used to complement the morphological analysis of parasites. Fish COI sequences were used to confirm the morphological identification of hosts [12, 13].

## Materials and Methods

### Fish sampling and identification

Fish were purchased at the fish markets in Sfax and Tunis, Tunisia and in Tripoli, Libya. These were previously caught by fishermen in the nearby coastal waters of the Mediterranean Sea. In all cases, the fish were dead when available for parasitological studies. No permits were required for the described study. Fish were identified morphologically according to keys [14] and books [15], and these identifications were challenged by analyses of COI sequences of individual fish (Table 1). Fish nomenclature follows [16] and [17].

**Table 1:** Host fish examined, their monogeneans and their COI sequences.

Host species	Fish specimen	Locality	Date	Fish COI, GenBank	Monogenean collected	Monogenean COI, GenBank
*M*. *marginata*	Mmargi3	Tunisia	25/09/2014	KX255749 *	*P*. *beverleyburtonae*	
*M*. *costae*	Mcostae1	Tunisia	13/06/2014	KX255750 *	*P*. *sosia*	KX255742 *
*M*. *costae*	Mcostae2	Tunisia	13/06/2014	KX255751 *	*P*. *sosia*	
*M*. *costae*	Mcostae3	Tunisia	15/04/2014	KT805240	*P*. *sosia*	
*M*. *costae*	Mcostae4	Libya	2013	-	*P*. *sosia*	
*M*. *costae*	Myco6	Tunisia	17/09/2015	KX255747 *	*P*. *sosia*	KX255741 * KX255743 * KX255744 *
*M*. *rubra*	Myru01	Libya	06/2013	KX255748 *	*P*. *regius*	
*M*. *rubra*	Myru02	Tunisia	10/09/2015	KU739518	*P*. *regius P*. *hayet* n. sp.	KX255745 * KX255746 *

*: new sequence.

### COI sequence of fish host

We used the QIAamp DNA Mini Kit (Qiagen), as per the manufacturer’s instructions, to perform DNA extraction. The 5′ region of the cytochrome oxidase I (COI) mitochondrial gene was amplified with the primers FishF1 (5′-TCAACCAACCACAAAGACATTGGCAC-3′) and FishR1 (5′-TAGACTTCTGGGTGGCCAAAGAATCA-3′) [12]. PCR reactions were performed in 20 μl, containing 1 ng of DNA, 1× CoralLoad PCR buffer, 3 mM MgCl_2_, 66 μM of each dNTP, 0.15 μM of each primer, and 0.5 units of Taq DNA polymerase (Qiagen). The amplification protocol was 4 min at 94°C, followed by 40 cycles at 94°C for 30 sec, 48°C for 40 sec, and 72°C for 50 sec, with a final extension at 72°C for 7 min. PCR products were purified and sequenced in both directions on a 3730xl DNA Analyzer 96-capillary sequencer (Applied Biosystems). We used CodonCode Aligner software (CodonCode Corporation, Dedham, MA, USA) to edit sequences, compared them to the GenBank database content with BLAST, and deposited them in GenBank under accession numbers KX255747 –KX255751 (Table 1). Species identification was confirmed with the BOLD identification engine [18]. Details about molecular identification of host fish are provided briefly in the description of the monogenean species.

### Monogenean morphology

Diplectanids collected from fish gills were prepared by three methods: a) mounted in ammonium picrate-glycerine [19] (designated as ‘p’); b) mounted in Berlese fluid (designated as ‘b’); and c) dehydrated in an ethanol series, stained with carmine and permanently mounted in Canada balsam (designated as ‘c’) [20]. Specimens were drawn using an Olympus BH2 microscope equipped with drawing apparatus and DIC optics. The terminology for the sclerotised parts, i.e. the male quadriloculate organ and the vagina follows Justine (2007) [1]. Measurements, in micrometres, were taken with the help of a custom-made transparent rule and are expressed as the mean followed in parentheses by the range, the standard deviation when n≥30, and (n) the number of observations; measurements were taken as in Fig 1 in Chaabane et al. (2015) [6]. The measurements of the right-hand haptoral hard-parts and left-hand equivalents were pooled. The measurements of the holotype are separated and indicated by ‘h’. Drawings were scanned and redrawn on a computer using Adobe Illustrator. The museum abbreviation used is as follows: MNHN, Muséum National d’Histoire Naturelle, Paris.

**Fig 1:**
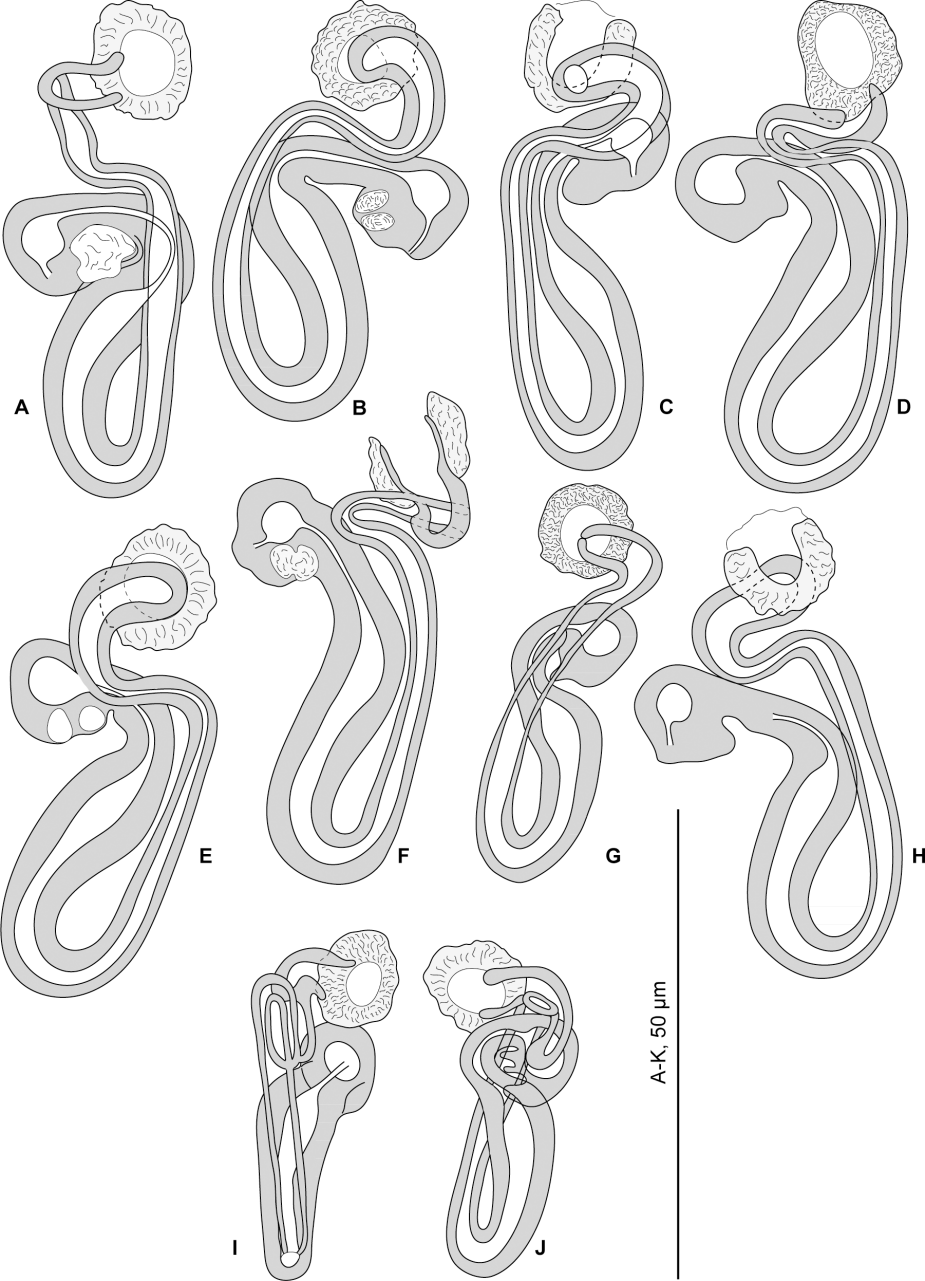
*Pseudorhabdosynochus beverleyburtonae* from *Mycteroperca marginata* in the Mediterranean Sea and Atlantic Ocean, various shapes of sclerotised vagina according to specimens, orientation and preparation. A-F, H, new specimens from Tunisia. G, MNHN HEL466, J, MNHN HEL465, voucher specimens from Brazil. I, MNHN 249H-Tc167, type specimen. A-F, H, Berlese. G, J, Gray and Wess medium. I, carmine.

### COI sequence of monogeneans

We used a QIAmp DNA Micro Kit (Qiagen) to extract DNA from whole monogenean specimens. The specific primers COI-ASmit1 (forward 5′-TTTTTTGGGCATCCTGAGGTTTAT-3′) and COI-ASmit2 (reverse 5′-TAAAGAAAGAACATAATGAAAATG-3′) were used to amplify a fragment of 424 bp of the COI gene [21]. The PCR reaction was performed in 20 μl, containing 1 ng of DNA, 1× CoralLoad PCR buffer, 3 mM MgCl_2_, 0.25 mM dNTP, 0.15 μM of each primer, and 0.5 units of Taq DNA polymerase (Qiagen). Thermocycles consisted of an initial denaturation step at 94°C for 2 min, followed by 37 cycles of denaturation at 94°C for 30 sec, annealing at 48°C for 40 sec, and extension at 72°C for 50 sec. The final extension was conducted at 72°C for 5 min. Sequences were edited with CodonCode Aligner software (CodonCode Corporation, Dedham, MA, USA), compared to the GenBank database content with BLAST, and deposited in GenBank under accession numbers KX255741 –KX255746 (Table 1).

Pairwise nucleotide distances were assessed using the Kimura 2-parameter (K2P) model [22] in MEGA 7. The phylogenetic tree was constructed using the Neighbour Joining (NJ) method based on the Kimura 2-parameter (K2P) model in MEGA 7 [23]; all codon positions were used.

### Nomenclatural acts

The electronic edition of this article conforms to the requirements of the amended International Code of Zoological Nomenclature, and hence the new names contained herein are available under that Code from the electronic edition of this article. This published work and the nomenclatural acts it contains have been registered in ZooBank, the online registration system for the ICZN. The ZooBank LSIDs (Life Science Identifiers) can be resolved and the associated information viewed through any standard web browser by appending the LSID to the prefix “http://zoobank.org/”. The LSID for this publication is: urn:lsid:zoobank.org:pub:CB13D383-7994-4BCE-BB8F-A46E874E26D3. The electronic edition of this work was published in a journal with an ISSN, and has been archived and is available from the following digital repositories: PubMed Central, LOCKSS.

## Results

### *Pseudorhabdosynochus beverleyburtonae* (Oliver, 1984) Kritsky & Beverley-Burton, 1986

Synonyms: *Diplectanum americanum* of Euzet & Oliver, 1965, *nec* Price, 1937; *Cycloplectanum americanum* (Price, 1937) Oliver, 1968 (pro parte); *Cycloplectanum beverleyburtonae* Oliver, 1984.

Type-host: Dusky grouper, *Mycteroperca marginata* (Lowe) (Perciformes, Epinephelidae) sometimes designated as *Epinephelus guaza* (Linnaeus) or *E*. *marginatus* (Lowe).

Molecular identification of fish via DNA barcoding: The COI sequence of our specimen (KX255749) (Table 1) was identical to three sequences (KU739519-521) previously obtained from the same fish species in the same locality (Tunisia) [24]. We conclude that our specimen belongs to *M*. *marginata*.

Site of infection: Gills

Type-locality: Off France, Mediterranean Sea, as “Côte Vermeille, Golfe du Lion, Méditerranée occidentale” [10].

Other localities: Off Rosas, Mediterranean Sea, France [25]; off Banyuls-sur-Mer, France [8, 9]; off Ilhas Cagarras, Rio de Janeiro, Brazil [11]; off Ubatuba, coast of São Paulo, south-eastern Brazil [26]; off Barra Velha, State of Santa Catarina, Brazil [2]; Sfax (fish market), Tunisia (present study). The Bay of Naples, Italy is sometimes indicated as a locality based on Ulmer & James (1981) [27] but this paper does not describe the sclerotised vagina so it could be, in our opinion, any species of *Pseudorhabdosynochus*.

Material examined: 2 type-specimens from off France (MNHN 249H-Tc167, 249H-Tc167bis) collected by Guy Oliver; voucher specimens from off Brazil (MNHN HEL465, HEL466, HEL470, HEL471) collected 3 February 2014 and deposited by Kritsky et al. [2] in the MNHN collection; new specimens collected off Tunisia (MNHN HEL560) (see Table 2).

**Table 2:** Measurements of *P*. *beverleyburtonae* from various sources.

Source	Santos, Buchmann & Gibson, 2000	Kritsky, Bakenhaster & Adams, 2015	Type-specimens, MNHN 249H-Tc167, 249H-Tc167 bis	Slides deposited in MNHN, HEL 465, HEL 466, HEL 470, HEL 471		MNHN HEL560, Present study
**Hosts**	*M*. *marginata*	*M*. *marginata*	*M*. *marginata*	*M*. *marginata*		*M*. *marginata*
**Locality**	Off Ilhas Cagarras, Rio de Janeiro, Brazil	Off Barra Velha, State of Santa Catarina, Brazil	Off Cap Béar, France	Off Barra Velha, State of Santa Catarina, Brazil		Off Sfax, Tunisia
**Method**	Gomori’s trichrome, Mayer’s paracarmine	Gomori’s trichrome, Gray and Wess medium	Carmine	Gomori’s trichrome	Gray and Wess medium	Berlese
**n**	26	**-**	2	2	2	17
**Body Length**	492–617	741 (569–974, n = 24)	510 (490–530, n = 2)	755 (710–800, n = 2)	780 (750–810, n = 2)	648 (570–750, n = 6)
**Body Width**	114–152	170 (143–235, n = 27)	185 (180–190, n = 2)	165 (130–200, n = 2)	165 (150–180, n = 2)	145 (120–180, n = 6)
**Haptor length**	61–95	-	-	-	-	-
**Haptor Width**	73–164	168 (141–197, n = 22)	150 (150–150, n = 2)	-	165 (150–180, n = 2)	-
**Pharynx Length**	34–48	-	44 (40–48, n = 2)	43 (34–52, n = 2)	44 (38–50, n = 2)	36 (29–40, n = 5)
**Pharynx Width**	32–41	47 (39–60, n = 31)	44 (40–48, n = 2)	45 (37–52, n = 2)	44 (38–50, n = 2)	32 (25–39, n = 5)
**Penis Internal Length**	46–82	-	62 (61–62, n = 2)	69 (67–70, n = 2)	68 (63–73, n = 2)	89 (82–100, n = 17)
**Penis Cone Length**	-	-	10 (9–11, n = 2)	12 (10–13, n = 2)	10 (8–11, n = 2)	11 (7–12, n = 17)
**Penis Tube Length**	27–55	-	-	-	33 (n = 2)	35 (29–44, n = 17)
**Penis Tube Diameter**	-	-	3 (n = 2)	-	4 (n = 2)	4 (4–4.5, n = 16)
**Penis (chamber + cone) Length**	-	84 (75–100, n = 28)	-	-	-	-
**Penis Filament Length**	-	-	-	-	34 (28–40, n = 2)	-
**Sclerotised Vagina Total Length**	34–41	-	39 (38–39, n = 2)	44 (43–44, n = 2)	40 (36–43, n = 2)	50 (45–54, n = 17)
**Primary Chamber External Diameter**	-	-	9 (8–9, n = 2)	7 (n = 2)	7 (n = 2)	9 (7–10, n = 17)
**Squamodisc Length**	-	56 (47–64, n = 32)	53 (50–60, n = 4)	54 (53–55, n = 2)	55 (54–55, n = 2)	-
**Squamodisc Width**	41–62	55 (42–67, n = 34)	51 (47–58, n = 4)	52 (51–53, n = 2)	51 (48–54, n = 2)	-
**Squamodisc, Number of Rows**	10–12	10–12 (usually 12)	10 (9–12, n = 4)	-	-	-
**Squamodisc, Number of Closed Rows**	2	2–3	2 (n = 4)	2 (n = 2)	-	-
**Ventral Anchor Outer Length**	39–55	56 (50–61, n = 15)	55 (53–58, n = 4)	55 (53–56, n = 2)	50 (n = 2)	60 (55–63, n = 15)
**Ventral Anchor Inner Length**	-	-	55 (53–57, n = 3)	55 (54–55, n = 2)	53 (52–53, n = 2)	58 (53–62, n = 15)
**Dorsal Anchor Outer Length**	34–52	49 (44–53, n = 16)	50 (50–51, n = 4)	49 (43–59, n = 3)	45 (n = 2)	50 (47–53, n = 14)
**Dorsal Anchor Inner Length**	-	-	33 (29–37, n = 3)	29 (20–33, n = 3)	31 (30–32, n = 2)	33 (31–39, n = 14)
**Ventral Bar Length**	50–78	77 (72–84, n = 13)	72 (68–75, n = 2)	-	72 (64–80, n = 2)	100 (93–106, n = 8)
**Ventral Bar Width**	-	-	15 (14–15, n = 2)	-	12 (n = 2)	18 (14–20, n = 8)
**Lateral (dorsal) Bar Length**	50–69	67 (61–72, n = 15)	64 (63–66, n = 4)	61 (54–74, n = 4)	59 (56–63, n = 4)	90 (84–96, n = 15)
**Lateral (dorsal) Bar Width**	-	-	20 (18–20, n = 4)	17 (14–22, n = 4)	16 (15–18, n = 4)	22 (17–26, n = 15)

Prevalence: In our newly examined fish specimen from Tunisia, 1/1.

#### Redescription (Figs 1 and 2)

**Fig 2:**
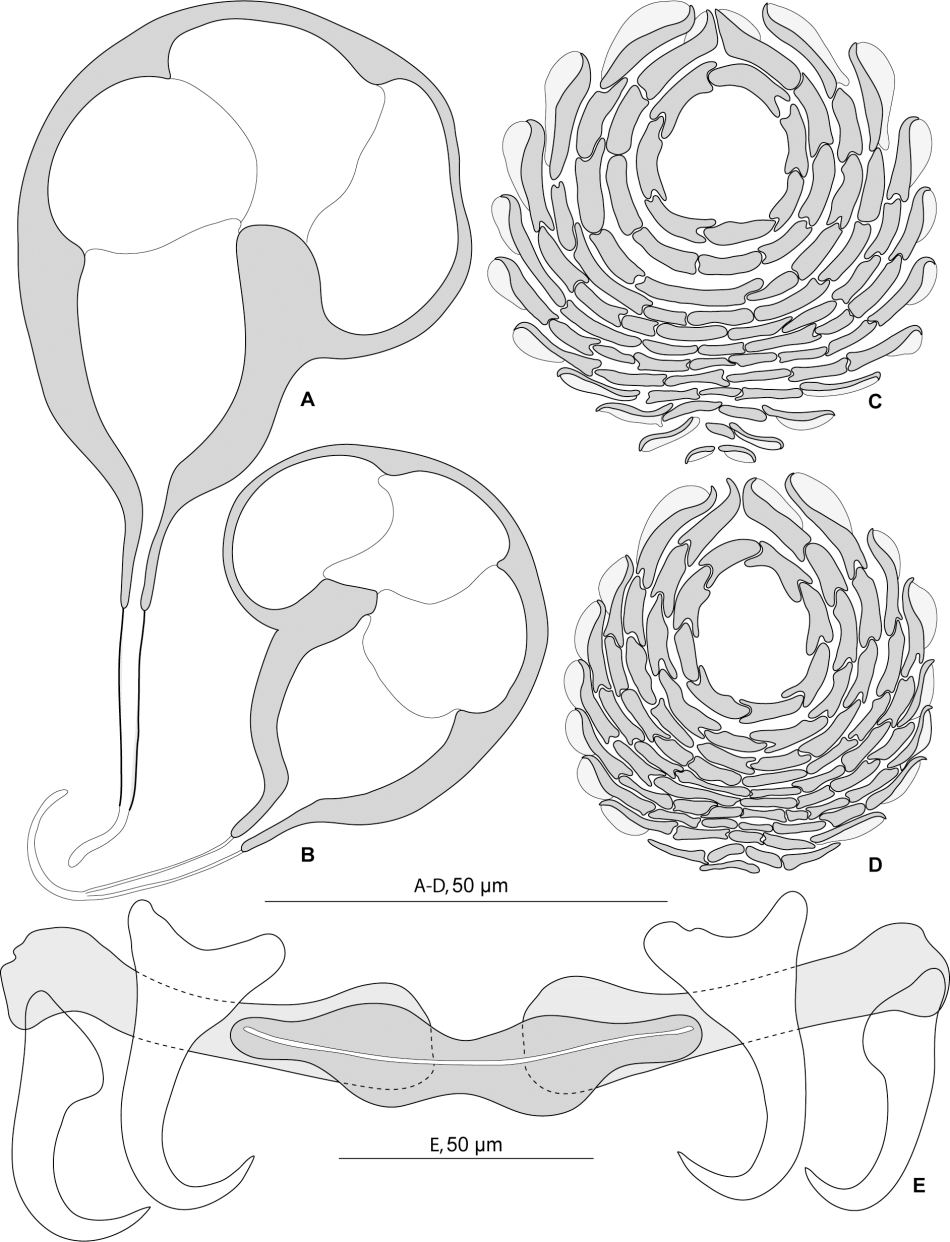
*Pseudorhabdosynochus beverleyburtonae* from *Mycteroperca marginata* in the Mediterranean Sea and Atlantic Ocean, quadriloculate organ, squamodiscs and haptoral hard parts. A, quadriloculate organ, MNHN 249H-Tc167, type-specimen. B, quadriloculate organ, voucher MNHN HEL466. C, ventral squamodisc, D, dorsal squamodisc, MNHN 249H-Tc167 bis, type-specimen. E, haptoral hard parts, new specimen from Tunisia. A, C, D, carmine. B, Gray and Wess medium. E, Berlese.

Measurements of 17 specimens in Berlese from Tunisia; for other specimens see Table 2. Body length b 648 (570–750, n = 6), including haptor; maximum width b 145 (120–180, n = 6) at level of ovary. Tegument smooth. Anterior region with 3 pairs of head organs and 2 pairs of dorsal eye-spots, distance between outer margins of anterior eye-spots b 25 (14–40, n = 16), of posterior eye-spots b 21 (14–37, n = 16). Pharynx median, subspherical, length b 36 (29–40, n = 5), width b 32 (25–39, n = 5). Haptor bearing 2 similar squamodiscs, 2 pairs of lateral anchors, 1 ventral bar and 2 lateral (dorsal) bars (Fig 2C–2E) and 14 marginal hooklets. Squamodiscs with 9–12 concentric rows of rodlets; two innermost rows forming circles (Fig 2C and 2D). Rodlets sometimes with visible spurs (‘éperons’). Ventral anchors with distinct handle and guard (Fig 2E), outer length b 60 (55–63, n = 15), inner length b 58 (53–62, n = 15). Dorsal anchors with indistinct guard (Fig 2E), outer length b 50 (47–53, n = 14), inner length b 33 (31–39, n = 14). Lateral (dorsal) bars, with flattened medial end (Fig 2E), length b 90 (84–96, n = 15), maximum width b 22 (17–26, n = 15). Ventral bar (Fig 2E), length b 100 (93–106, n = 8), maximum width, b18 (14–20, n = 8). Male copulatory organ a quadriloculate organ, first (anterior) chamber almost as sclerotised as the 3 others; fourth chamber forming short cone, prolonged by thin sclerotised tube; inner length b 89 (82–100, n = 17); cone length b 11 (7–12, n = 17); tube length b 35 (29–44, n = 17); tube diameter b 4 (4–4.5, n = 16). Filament of variable length (Fig 2A and 2B).

*Sclerotised vagina* comprises sclerotised trumpet, primary canal, primary chamber, secondary canal and secondary chamber. Trumpet anterior, ring-shaped; primary canal with regular diameter and medium-thick wall, curved in its anterior part, sometimes looped; connection between primary canal and primary chamber posterior; primary chamber elongate, roughly parallel to primary canal but reversely oriented, its wall slightly thicker than primary canal; secondary canal well visible, often curved, its lumen thin, its wall slightly less sclerotised than chambers; secondary chamber roughly spherical, its wall thick, smaller than primary chamber; secondary chamber located just posterior to trumpet, often at the level of curve of primary canal (Fig 1A–1J). Total length of sclerotised vagina b 50 (45–54, n = 17). External diameter of primary chamber b 9 (7–10, n = 17).

#### Comments

*Pseudorhabdosynochus beverleyburtonae* was originally described by Oliver as *Cycloplectanum beverleyburtonae*, from *M*. *marginata* (as *Epinephelus guaza*) in the Western Mediterranean Sea off France [10]. Kritsky & Beverley-Burton (1986) regarded *Cycloplectanum* Oliver, 1968 a junior synonym of *Pseudorhabdosynochus* Yamaguti, 1958 and placed several species, including *C*. *beverleyburtonae*, in the latter genus [28]. Most subsequent authors have accepted *Pseudorhabdosynochus* as the valid genus; however, Oliver continued to designate the species as *C*. *beverleyburtonae* [25].

*Pseudorhabdosynochus beverleyburtonae* parasitises *M*. *marginata*, which is a trans-Atlantic grouper fish. The original description does not include any measurements [10]. Measurements in specimens from various origins show differences (Table 2) but these should be attributed to various methods of fixation and degree of flattening, as is usual in diplectanids [20]. The morphology of the sclerotised vagina is similar in all specimens, including our newly examined specimens from Tunisia. We conclude, as previous authors [2, 11], that the populations assigned to *M*. *marginata* from the Mediterranean Sea and both sides of the Atlantic Ocean are conspecific. Tunisia is a new geographical record for the species, and is the first record on the southern shore of the Mediterranean Sea.

#### Differential diagnosis

*Pseudorhabdosynochus beverleyburtonae* is close to *P*. *oliveri* n. sp., *P*. *sosia* and *P*. *hayet* n. sp. in terms of general morphology of body, presence of two circular innermost rows of rodlets in squamodiscs and general morphology of the sclerotised vagina. It is separated from *P*. *oliveri*, which also occurs on *M*. *marginata*, by the shape of its primary canal (narrow in *P*. *beverleyburtonae* vs. wide in *P*. *oliveri*), and from *P*. *sosia* and *P*. *hayet* by the shape of its primary chamber, wider and more sclerotised in the two latter species.

### Pseudorhabdosynochus oliveri n. sp.

urn:lsid:zoobank.org:act:8BDBF329-0700-44A1-B720-6C172ECE0701

Type-host: *Mycteroperca marginata* (Lowe) (Perciformes, Epinephelidae)

Site of infection: Gills

Type-locality: Off Cap Béar, Mediterranean Sea, France; date 4 February 1965.

Material examined: 4 voucher specimens MNHN HEL68 OLI 8–1 to 8–4 on slides, labelled as *Cycloplectanum beverleyburtonae* by Guy Oliver.

Holotype: MNHN HEL68 OLI 8–2

Paratypes: MNHN HEL68 OLI 8–1, HEL68 OLI 8–3, HEL68 OLI 8–4

Etymology: This species is named for Guy Oliver, French parasitologist, in recognition of his research on fish monogeneans.

#### Description (Figs 3 and 4)

**Fig 3:**
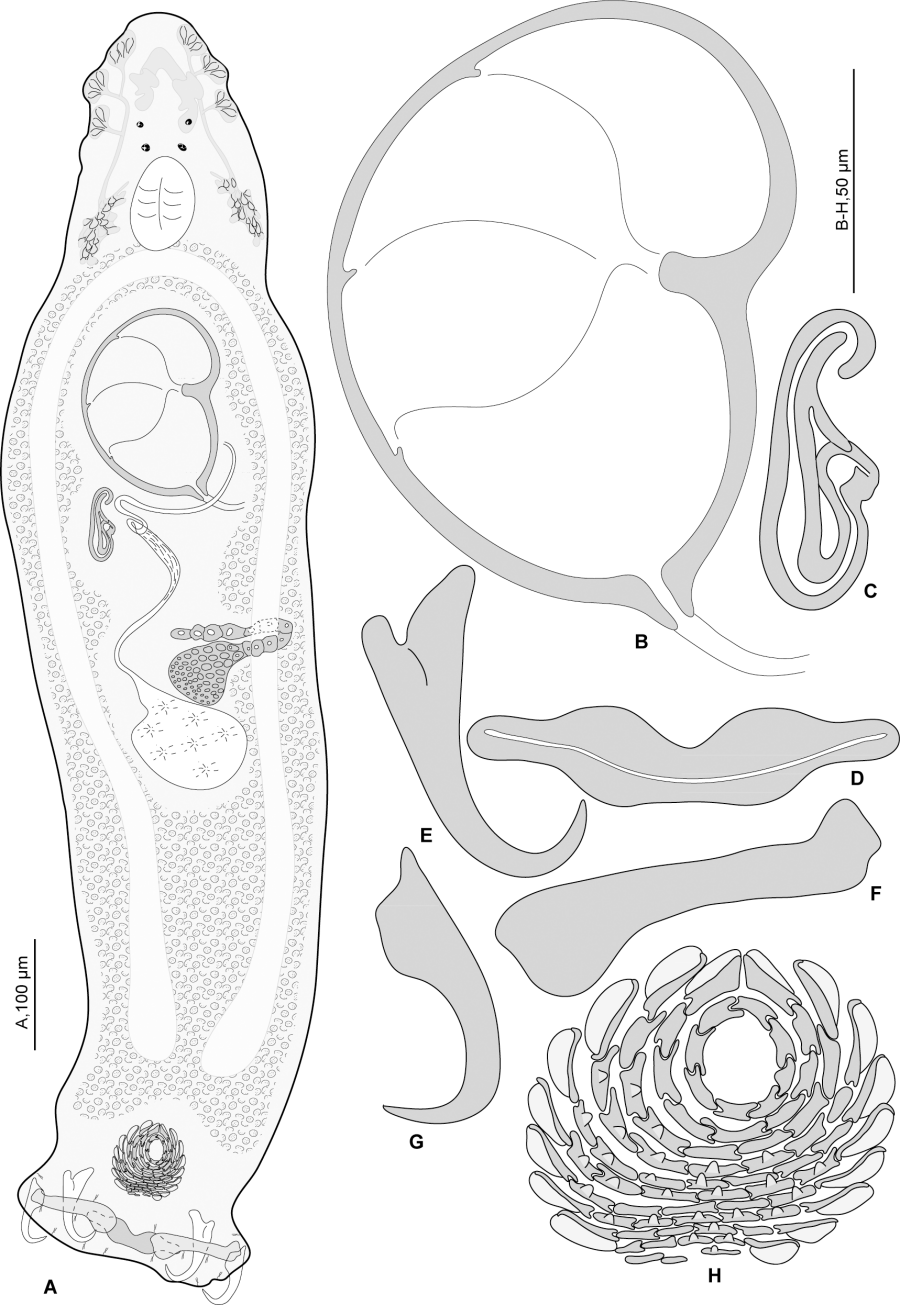
*Pseudorhabdosynochus oliveri* n. sp. from *Mycteroperca marginata* off France. A, composite, dorsal view. B, male quadriloculate organ. C, vagina. D, ventral bar. E, ventral anchor. F, lateral (dorsal) bar. G, dorsal anchor. H, dorsal squamodisc. All in carmine.

Measurements of 4 specimens in carmine. Body length h 1100, c 1050 (1000–1100, n = 2), including haptor; maximum width h 250, c 235 (220–250, n = 2) at level of ovary (Fig 3A). Tegument smooth. Anterior region with 3 pairs of head organs and 2 pairs of dorsal eye-spots, distance between outer margins of anterior eye-spots h 50, c 42 (35–50, n = 3), of posterior eye-spots h 40, c 36 (30–40, n = 3). Pharynx median, ovate, length c 56 (50–62, n = 2), width c 59 (56–62, n = 2). Oesophagus very short or absent. Two simple lateral intestinal caeca not united posteriorly. Haptor bearing 2 squamodiscs, 2 pairs of lateral anchors, 1 ventral bar and 2 lateral (dorsal) bars, and 14 marginal hooklets. Squamodiscs with 10–11 concentric rows of rodlets; the 2 innermost rows form circles. Rodlets with visible spurs (‘éperons’) (Fig 3H). Squamodisc length c 72 (n = 2), width c 85 (81–88, n = 2). Ventral anchors with distinct handle and guard (Fig 3E), outer length h 70, c 72 (67–82, n = 8), inner length h 73, c 73 (70–78, n = 8). Dorsal anchors with indistinct guard (Fig 3G), outer length c 66 (63–73, n = 6), inner length c 47 (43–50, n = 5). Lateral (dorsal) bars, with flattened medial end (Fig 3F), length h 85, c 87 (84–91, n = 8), maximum width h 24, c 24 (23–25, n = 8). Ventral bar with constricted median portion (Fig 3D), length h 104, c 102 (97–105, n = 4), maximum width, h 20, c 20 (16–22, n = 4). Testis subspherical, posterior, intercaecal. Male copulatory organ a quadriloculate organ, first (anterior) chamber as sclerotised as the 3 others; fourth chamber forming short cone, prolonged by thin sclerotised tube. Inner length c 133 (128–138, n = 2); cone length c 9 (8–10, n = 2); tube length c 35 (33–36, n = 2); tube diameter c 6 (5–6, n = 2). Filament not seen (Fig 3B).

**Fig 4:**
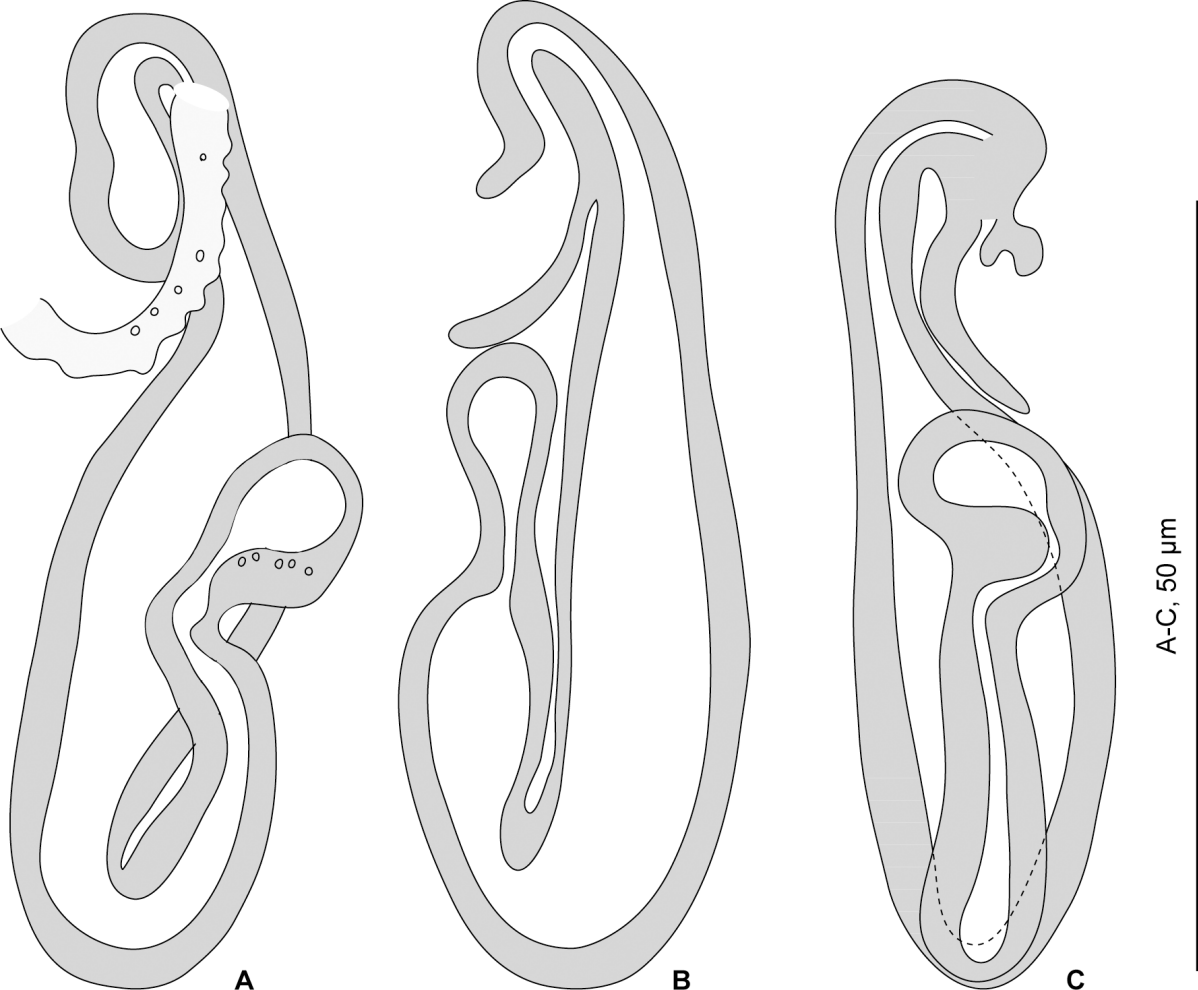
*Pseudorhabdosynochus oliveri* n. sp. from *Mycteroperca marginata* off France, different shapes of sclerotised vagina according to specimens and orientation. A, MNHN HEL68 OLI 8–1. B, MNHN HEL68 OLI 8–3. C, MNHN HEL68 OLI 8–4. All in carmine.

Vitelline follicles lateral, coextensive with intestinal caeca and contiguous posterior to testis. Ovary on right side, looping dorsoventrally around right intestinal caecum.

*Sclerotised vagina* comprises sclerotised trumpet, primary canal, primary chamber, secondary canal and secondary chamber. Trumpet in anterior part; primary canal with thick wall, curved in its anterior part, enlarged in its posterior part with wide lumen; connection between primary canal and primary chamber posterior; primary chamber elongate, roughly parallel to primary canal but reversely oriented, its wall the same thickness as primary canal; secondary canal short, its lumen thin; secondary chamber located just posterior to trumpet, roughly spherical, its wall thick, smaller than primary chamber (Figs 3C and 4A–4C). Total length of sclerotised vagina c 63 (59–65, n = 4). Diameter of primary canal h 11, c 14 (11–16, n = 4).

#### Comments

This new species was found among voucher specimens labelled as *Cycloplectanum beverleyburtonae* by Guy Oliver (now *P*. *beverleyburtonae*). These specimens were deposited by Guy Oliver in the MNHN collections after his retirement and were not mentioned in the original description. This is not the first time that new species of *Pseudorhabdosynochus* are found among museum specimens labelled as other species; previous cases include *P*. *satyui* Justine, 2009, found among specimens of *P*. *epinepheli* Yamaguti, 1958 [29], *P*. *williamsi* Kritsky et al., 2015 found among specimens of *P*. *monaensis* Dyer et al., 1994, and *P*. *justinella* Kritsky et al., 2015 found among specimens of *P*. *yucatanensis* Vidal-Martinez et al., 1997. In the latter case, Kritsky et al. (2015) demonstrated that the original description of *P*. *yucatanensis* [30] contained drawings of specimens from both species; in the case of the *P*. *beverleyburtonae/P*. *oliveri* couple, we did not find any indication of *P*. *oliveri* being included in the original description of *P*. *beverleyburtonae*.

Although *P*. *oliveri* and *P*. *beverleyburtonae* apparently co-occurred on dusky groupers from off France examined by Oliver, we did not find the former in our fish specimen from Tunisia.

#### Differential diagnosis

*Pseudorhabdosynochus oliveri* is close to *P*. *beverleyburtonae*, *P*. *sosia* and *P*. *hayet* n. sp. in terms of general morphology of body, presence of two circular innermost rows of rodlets in squamodiscs and general morphology of the sclerotised vagina. It is separated from the three others by the development of its primary canal, which is wide, vs. thin, and the small primary chamber, vs. large in the other three species.

### Pseudorhabdosynochus sosia Neifar & Euzet, 2007

Type-host: Goldblotch grouper, *Mycteroperca costae* (Steindachner) (Perciformes, Epinephelidae); synonyms: *Epinephelus alexandrinus* (Valenciennes), *Epinephelus costae*.

Molecular identification of fish via DNA barcoding: Four specimens from Tunisia were sequenced (Table 1), including three new and one already sequenced as KT805240 [31]. All sequences had 99–100% similarity with sequences already identified as *M*. *costae* in GenBank, confirming the morphological identification of the host.

Site of infection: Gills

Type-locality: Off Sfax, Tunisia.

Other localities: Off Zarzis (Tunisia) [3]; off Dakar (Senegal) [3]; Tripoli (fish market), Libya (present study).

Material examined: Holotype, MNHN HEL11-Th84 (darkened picrate slide); paratypes, MNHN HEL12-Th85, MNHN HEL13-Th86 (paratype specimens marked on slides); voucher specimens collected by Neifar and Euzet (MNHN HEL561) from off Tunisia; new specimens collected from off Tunisia (MNHN HEL562) (Table 3).

**Table 3:** Measurements of *P*. *sosia* and *P*. *hayet* n. sp.

Species	*P*. *sosia* Neifar & Euzet, 2007				*P*. *hayet* n. sp.		
Hosts	*M*. *costae*				*M*. *rubra*		
**Source**	**Neifar and Euzet, 2007**	**Paratypes**, MNHN HEL12-Th 85, MNHN HEL13-Th 86	**MNHN HEL561**, Vouchers collected by Neifar & Euzet	**MNHN HEL562**, Vouchers, newly collected specimens	**MNHN HEL563-564**, Specimens collected by Neifar & Euzet	**MNHN HEL565**, Specimens collected by Neifar	**MNHN 306HG**, Specimens from Euzet collection
**Locality**	Off Sfax, Tunisia	Off Sfax, Tunisia	Off Sfax and Zarzis, Tunisia	Off Sfax, Tunisia	Off Dakar, Senegal	Off Sfax, Tunisia	Off Dakar, Senegal
**Methods**	Picrate-glycerine, carmine, Berlese	Picrate-glycerine	Picrate-glycerine	Berlese	Carmine	Picrate-glycerine	Berlese
**n**	34	9	19	30	4	3	5
**Body Length**	1,100 (800–1,300, n = 24)	398 (347–438, n = 9)	733 (550–950, n = 17)	1,039 (800–1,200, n = 12)	795 (710–920, n = 4)	970 (830–1,110, n = 2)	1,150 (1,100–1,200, n = 2)
**Body Width**	270 (170–350, n = 24)	79 (75–91, n = 9)	150 (120–180, n = 16)	219 (150–330, n = 11)	215 (180–260, n = 4)	180 (130–230, n = 2)	245 (240–250, n = 2)
**Haptor Width**	-	85 (75–98, n = 9)	147 (130–166, n = 13)	198 (180–210, n = 4)	165 (140–180, n = 4)	-	-
**Pharynx Length**	66 (50–80, n = 14)	28 (18–42, n = 9)	38 (25–50, n = 18)	-	46 (42–48, n = 4)	-	-
**Pharynx Width**	57 (40–70, n = 14)	24 (18–30, n = 9)	34 (25–40, n = 18)	-	49 (45–52, n = 4)	-	-
**Penis Internal Length**	110±3 (90–130, n = 29)	**84** (75–91, n = 8)	**81** (65–94, n = 15)	**92** (63–120, n = 23)	**85** (83–92, n = 4)	**89** (86–94, n = 3)	**150** (140–165, n = 5)
**Penis Cone Length**	13±1 (10–17, n = 29)	11 (8–15, n = 8)	8 (6–10, n = 14)	8 (5–10, n = 26)	7 (5–9, n = 4)	7 (6–7, n = 2)	10 (n = 5)
**Penis Tube Length**	38±2 (25–45, n = 29)	29 (24–42, n = 6)	23 (10–27, n = 9)	30 (24–39, n = 27)	18 (15–20, n = 2)		26 (5–33, n = 5)
**Penis Tube Diameter**	-	4 (4–4, n = 8)	4 (3–4, n = 13)	3 (3–4, n = 27)	4 (3–5, n = 4)	4 (n = 2)	5 (4–5, n = 3)
**Penis Filament Length**	-	-	-	28 (20–33, n = 3)	30 (n = 2)	-	-
**Sclerotised Vagina Total Length**	48±1 (40–55, n = 34)	**35** (32–38, n = 9)	**36** (26–40, n = 19)	**44**±4.3 (35–58, n = 30)	**46** (45–49, n = 4)	**43** (40–45, n = 3)	**61** (54–70, n = 5)
**Primary Chamber External Diameter**	-	12 (11–14, n = 9)	13 (10–15, n = 19)	15 (11–17, n = 28)	10 (n = 4)	9 (7–12, n = 3)	15 (14–16, n = 5)
**Squamodisc Length**		46 (44–49, n = 7)	51 (40–62, n = 15)	63 (52–80, n = 23)	53 (40–62, n = 8)	49 (48–50, n = 2)	-
**Squamodisc Width**	67 (60–80, n = 12)	44 (39–45, n = 7)	45 (29–51, n = 14)	62 (53–73, n = 23)	52 (38–58, n = 8)	63 (62–63, n = 2)	-
**Squamodisc, Number of Rows**	10–11	11 (10–11, n = 5)		11 (10–12, n = 22)	10 (10–11, n = 4)	10 (n = 1)	-
**Squamodisc, Number of Closed Rows**	1–2	2 (n = 5)	2 (n = 11)	2 (n = 22)	2 (n = 2)	-	-
**Ventral Anchor Outer Length**	60±1 (55–67, n = 34)	41 (36–45, n = 14)	44 (41–48, n = 27)	48±3 (42–53, n = 33)	49 (45–52, n = 2)	46 (43–48, n = 4)	51 (48–55, n = 8)
**Ventral Anchor Inner Length**	-	41 (39–45, n = 13)	44 (38–50, n = 28)	47±5.2 (40–74, n = 35)	48 (45–50, n = 6)	45 (40–48, n = 4)	48 (38–52, n = 8)
**Dorsal Anchor Outer Length**	52 (50–57, n = 28)	37 (33–41, n = 15)	38±2 (35–41, n = 29)	40±2.1 (3–43, n = 38)	40 (38–42, n = 7)	42 (40–43, n = 4)	43 (41–45, n = 7)
**Dorsal Anchor Inner Length**	-	24 (23–27, n = 13)	24±1.4 (22–28, n = 29)	25±2.4 (21–32, n = 36)	25 (24–26, n = 7)	24 (23–26, n = 4)	26 (25–30, n = 7)
**Ventral Bar Length**	83±3 (70–105, n = 34)	70 (57–128, n = 7)	61 (55–73, n = 15)	88 (68–100, n = 20)	69 (63–73, n = 4)	77 (68–86, n = 2)	93 (85–100, n = 3)
**Ventral Bar Width**	12±1 (7–17, n = 34)	8 (7–10, n = 7)	10 (7–13, n = 15)	16 (13–20, n = 20)	13 (n = 4)	11 (n = 2)	18 (17–20, n = 3)
**Lateral Bar Length**	66±2 (58–73, n = 36)	47 (45–49, n = 18)	50±4.2 (42–63, n = 33)	72±4.7 (60–80, n = 40)	53 (47–55, n = 8)	54 (53–56, n = 4)	75 (72–78, n = 8)
**Lateral Bar Width**	14±2 (12–22, n = 36)	12 (9–15, n = 18)	13±1 (10–15, n = 33)	21±1.9 (17–25, n = 40)	16 (13–18, n = 8)	15 (14–16, n = 4)	26 (17–30, n = 8)

Bold: important differences for species differentiation.

Prevalence: In our specimens from Tunisia, 4/4 (100%); from Libya, 1/1 (100%).

Molecular characterisation: COI sequences were obtained for 4 specimens from Tunisia (Table 1).

#### Redescription (Fig 5)

**Fig 5:**
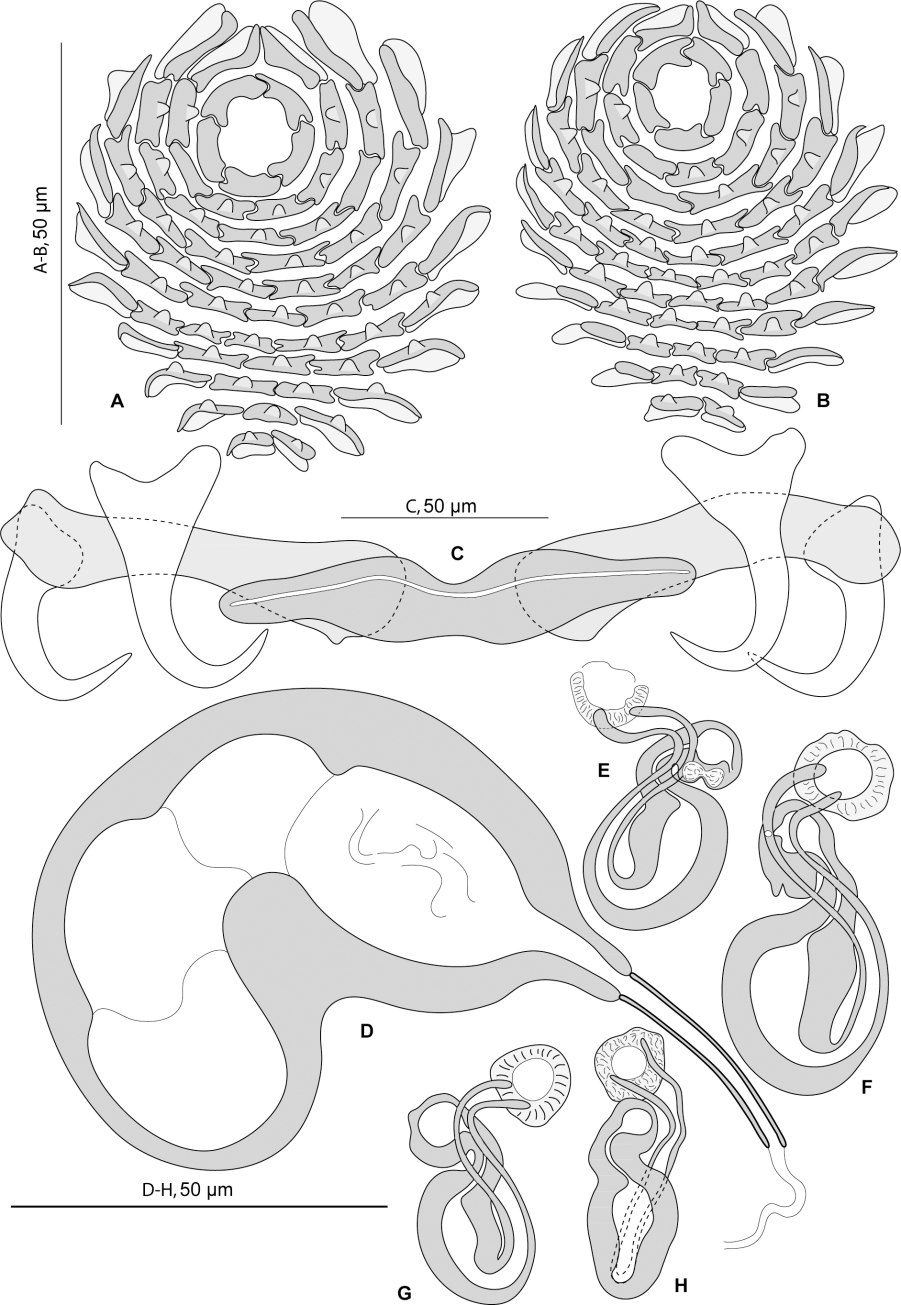
*Pseudorhabdosynochus sosia* from *Mycteroperca costae* in the Mediterranean Sea and Eastern Atlantic Ocean, sclerotised parts. A, ventral squamodisc. B, dorsal squamodisc. C, haptor hard parts. D, quadriloculate organ. E, F, vaginae. A-F, newly collected specimens from Tunisia, MNHN HEL562. G, H, vaginae, voucher specimens MNHN HEL561 from Tunisia. A-F, Berlese. G, H, picrate-glycerine.

Measurements of 30 specimens in Berlese. Body length b 1,039 (800–1,200, n = 12), including haptor; maximum width b 219 (150–330, n = 11) at level of ovary. Tegument smooth. Anterior region with 3 pairs of head organs and 2 pairs of dorsal eye-spots, distance between outer margins of anterior eye-spots b 39 (27–63, n = 22), of posterior eye-spots b 30 (15–38, n = 26). Pharynx median, subspherical. Oesophagus very short or absent. Two simple lateral intestinal caeca not united posteriorly. Haptor bearing 2 squamodiscs, 2 pairs of lateral anchors, 1 ventral bar and 2 lateral (dorsal) bars and 14 marginal hooklets; width b 198 (180–210, n = 4). Squamodiscs with 10–12 concentric rows of rodlets; 2 innermost rows forming circles. Rodlets with visible spurs (‘éperons’) (Fig 5A and 5B). Squamodisc length b 63 (52–80, n = 23), width b 62 (53–73, n = 23). Ventral anchors with distinct handle and guard (Fig 5C), outer length b 48±3 (42–53, n = 33), inner length b 47±5.2 (40–74, n = 35). Dorsal anchors with indistinct guard (Fig 5C), outer length b 40±2.1 (33–43, n = 38), inner length b 25±2.4 (21–32, n = 36). Lateral (dorsal) bars with wide flattened medial extremity and cylindrical lateral extremity (Fig 5C), length b 72±4.7 (60–80, n = 40), width b 21±1.9 (17–25, n = 40). Ventral bar (Fig 5C), length b 88 (68–100, n = 20), width b 16 (13–20, n = 20). Testis subspherical, posterior, intercaecal. Male copulatory organ a quadriloculate organ, length b 92 (63–120, n = 23); divided into four chambers, fourth chamber more sclerotised than 3 anterior chambers; fourth chamber ends in short sclerotised cone, length b 8 (5–10, n = 26), prolonged by sclerotised tube, length b 30 (24–39, n = 27), diameter b 3 (3–4, n = 27); end of tube prolonged by thin unsclerotised filament, length b 28 (20–33, n = 3) (Fig 5D).

*Sclerotised vagina* comprises sclerotised trumpet, primary canal, primary chamber, secondary canal and secondary chamber. Trumpet anterior, ring-shaped; primary canal with regular diameter and medium-thick wall, curved or S-shaped; connection between primary canal and primary chamber posterior; primary chamber ovoid or pear-shaped, roughly parallel to primary canal but reversely oriented, its wall thick; secondary canal well visible, its lumen thin, its wall less sclerotised than chambers; secondary chamber spherical, its wall thick, smaller than primary chamber; secondary chamber located just posterior to trumpet (Fig 5E–5H). Sclerotised vagina length b 44±4.3 (35–58, n = 30), external diameter of primary chamber b 15 (11–17, n = 28).

#### Differential diagnosis

By the morphology of its sclerotised vagina, *P*. *sosia* is close to *P*. *beverleyburtonae*, *P*. *oliveri* n. sp. and *P*. *hayet* n. sp. Indeed, Neifar & Euzet named the species for its resemblance with *P*. *beverleyburtonae* [3]. *Pseudorhabdosynochus sosia* and *P*. *beverleyburtonae* have similar vaginal lengths but can be differentiated, as indicated by Neifar & Euzet, 2007, by the external diameter of the primary chamber (larger in *P*. *sosia*) and its shape (pear-shaped in *P*. *sosia* vs elongated in *P*. *beverleyburtonae*), and by the shape and length of the primary canal (longer and more coiled in *P*. *beverleyburtonae*). *Pseudorhabdosynochus sosia* is readily distinguished from *P*. *oliveri* by the shape of its primary canal (thin vs. wide in the latter).

The morphology of the vagina is very similar in *P*. *sosia* and *P*. *hayet*, but *P*. *sosia* differs by smaller male copulatory organ (see details under *P*. *hayet*, and Table 3), different host (*M*. *costae* for *P*. *sosia* vs *M*. *rubra* for *P*. *hayet*) (Table 1), and divergent COI sequences (Table 4).

**Table 4:** Estimates of evolutionary divergence between COI sequences of *Pseudorhabdosynochus* spp.

Species	Accession number		[1]	[2]	[3]	[4]	[5]	[6]	[7]	[8]
*Pseudorhabdosynochus hayet* n. sp	KX255746	[1]								
*Pseudorhabdosynochus sosia*	KX255744	[2]	**0.13**							
*Pseudorhabdosynochus sosia*	KX255743	[3]	**0.14**	0.00						
*Pseudorhabdosynochus sosia*	KX255741	[4]	**0.14**	0.00	0.00					
*Pseudorhabdosynochus sosia*	KX255742	[5]	**0.13**	0.00	0.00	0.00				
*Pseudorhabdosynochus regius*	KX255745	[6]	0.26	0.21	0.21	0.21	0.21			
*Pseudorhabdosynochus sulamericanus*	KT023569	[7]	0.21	0.19	0.20	0.20	0.19	0.22		
*Pseudorhabdosynochus cyanopodus*	JQ400135	[8]	0.26	0.20	0.20	0.20	0.20	0.23	0.19	
*Lamellodiscus ignoratus*	JF427655	[9]	0.39	0.34	0.34	0.34	0.34	0.31	0.38	0.38

The analysis involved 9 nucleotide sequences, and there were a total of 290 positions in the final dataset. Bold: differences between *P*. *hayet* n. sp. and *P*. *sosia*.

### Pseudorhabdosynochus hayet n. sp.

urn:lsid:zoobank.org:act:10C2CE08-E772-4817-A4BC-EF7CF91ED44A

Synonym: *Pseudorhabdosynochus* sp. of Chaabane et al., 2015 (Table 1 in [6])

Type-host: Mottled grouper *Mycteroperca rubra* (Bloch) (Perciformes, Epinephelidae)

Molecular identification of fish via DNA barcoding: Two fish were sequenced, one from Libya (new sequence KX255748) and one from Tunisia (sequence already published as KU739518 [24] (Table 1). The sequences were similar (99% identity) to sequences of the same fish species, therefore confirming the identity of the host fish.

Site of infection: Gills.

Type-locality: Off Dakar, Senegal.

Other localities: Sfax and Tunis (fish markets), Tunisia (present study)

Material examined: Specimens off Sfax and Dakar (Table 3).

Specimens deposited: Holotype, MNHN HEL563 (carmine), from off Senegal, material collected by Neifar & Euzet, precise date unknown (2002–2004); paratypes MNHN HEL564 from off Senegal (carmine), material collected by Neifar & Euzet, precise date unknown (2002–2004); paratypes MNHN 306HG from off Senegal (Berlese), material collected by Neifar & Euzet (2004) in Euzet’s collection; paratypes MNHN HEL565 from off Tunisia collected by Neifar (picrate). The holotype was chosen among carmine slides because slides in Canada balsam are permanent. One specimen collected off Tunis, Tunisia, used for molecular analysis and destroyed.

Prevalence: In Tunisia, 1/1, in Libya, 0/1.

Molecular characterisation: A COI sequence was obtained for a specimen from off Tunis, Tunisia (Table 1).

Etymology: The specific name, *hayet*, is from Arabic (*حياة*). The species is named after the mother of the first author. Name in apposition, invariable.

#### Description (Figs 6 and 7)

**Fig 6:**
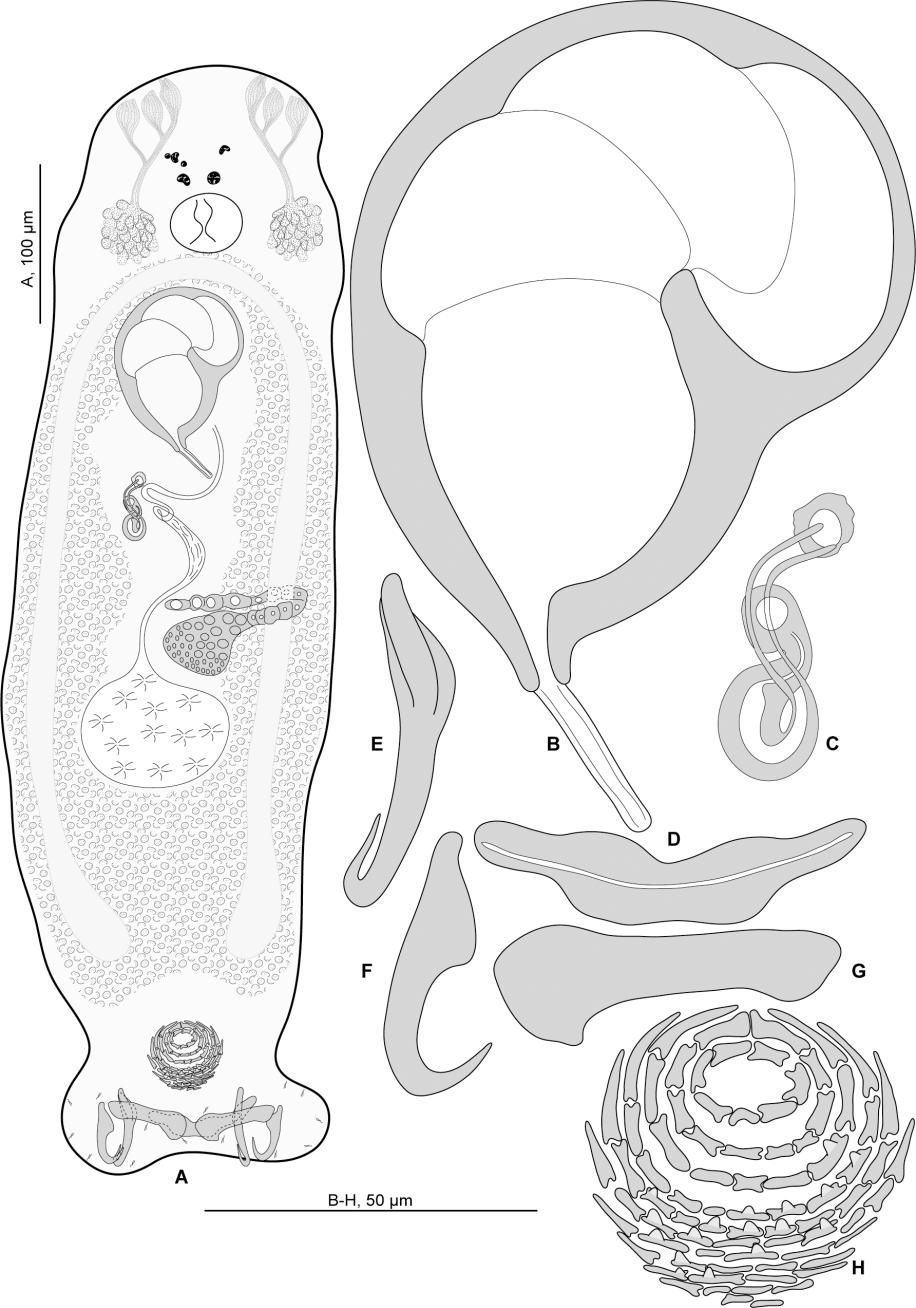
*Pseudorhabdosynochus hayet* n. sp. from *Mycteroperca rubra* off Senegal. A, holotype, dorsal view. B, male quadriloculate organ. C, vagina. D, ventral bar. E, ventral anchor. F, dorsal anchor. G, lateral (dorsal) bar. H, ventral squamodisc. All in carmine.

Measurements based on 12 specimens in picrate, Berlese and carmine; holotype in carmine. Body length h 730, c 795 (710–920, n = 4), p 970 (830–1,110, n = 2), b 1,150 (1,100–1,200, n = 2) including haptor; maximum width h 200, c 215 (180–260, n = 4), b 245 (240–250, n = 2), at level of ovary (Fig 6A). Tegument smooth. Anterior region with 3 pairs of head organs and 2 pairs of dorsal eye-spots, distance between outer margins of anterior eye-spots h 42, c 39 (32–43, n = 4), b 51 (30–62, n = 3), of posterior eye-spots h 28, c 28 (28–29, n = 4), b 32 (20–40, n = 3). Pharynx median, subspherical length h 42, c 46 (42–48, n = 4), width c 49 (45–52, n = 4). Oesophagus very short or absent. Two simple lateral intestinal caeca not united posteriorly. Haptor bearing 2 squamodiscs, 2 pairs of lateral anchors, 1 ventral bar and 2 lateral (dorsal) bars and 14 marginal hooklets; width h 170, c 165 (140–180, n = 4). Squamodiscs with 10–11 concentric rows of rodlets; 2 innermost rows forming circle. Rodlets with visible spurs (‘éperons’) (Fig 6H). Squamodisc length h 50, p 49 (48–50, n = 2), c 53 (40–62, n = 8), width h 51, p 63 (62–63, n = 2), c 52 (38–58, n = 8). Ventral anchors with distinct handle and guard (Fig 6E), outer length c 49 (45–52, n = 2), p 46 (43–48, n = 4), b 51 (48–55, n = 8), inner length h 46, c 48 (45–50, n = 6), p 45 (40–48, n = 4), b 48 (38–52, n = 8). Dorsal anchors with indistinct guard (Fig 6F), outer length h 39, c 40 (38–42, n = 7), p 42 (40–43, n = 4), b 43 (41–45, n = 7), inner length h 26, c 25 (24–26, n = 7), p 24 (23–26, n = 4), b 26 (25–30, n = 7). Lateral (dorsal) bars with wide flattened medial extremity and cylindrical lateral extremity (Fig 6G), length h 55, c 53 (47–55, n = 8), p 54 (53–56, n = 4), b 75 (72–78, n = 8), width h 18, c16 (1–18, n = 8), p 15 (14–16, n = 4), b 26 (17–30, n = 8). Ventral bar with blunt extremities (Fig 6D), length h 73, c 69 (63–73, n = 4), b 93 (85–100, n = 3), width h 13, c 13 (n = 4), b 18 (17–20, n = 3). Testis subspherical, posterior, intercaecal. Male copulatory organ a quadriloculate organ, length h 92, c 85 (83–92, n = 4), p 89 (86–94, n = 3), b 150 (140–165, n = 5); divided into four chambers, fourth (posterior) chamber more sclerotised than 3 anterior chambers, finishes with short sclerotised cone, cone length h 7, c 7 (5–9, n = 4), p 7 (6–7, n = 2), b 10 (n = 5) prolonged by sclerotised tube, tube length c 18 (15–20, n = 2), b 26 (5–33, n = 5), tube diameter h 4.5, c 4 (3–5, n = 4), p 4 (n = 2), b 5 (4–5, n = 3); end of tube sometimes prolonged by thin unsclerotised filament, length c 30 (n = 2) (Fig 6B). Ovary subequatorial, intercaecal, pre-testicular, encircles right caecum. Vitelline follicles present from pre-pharynx level to haptoral peduncle.

**Fig 7:**
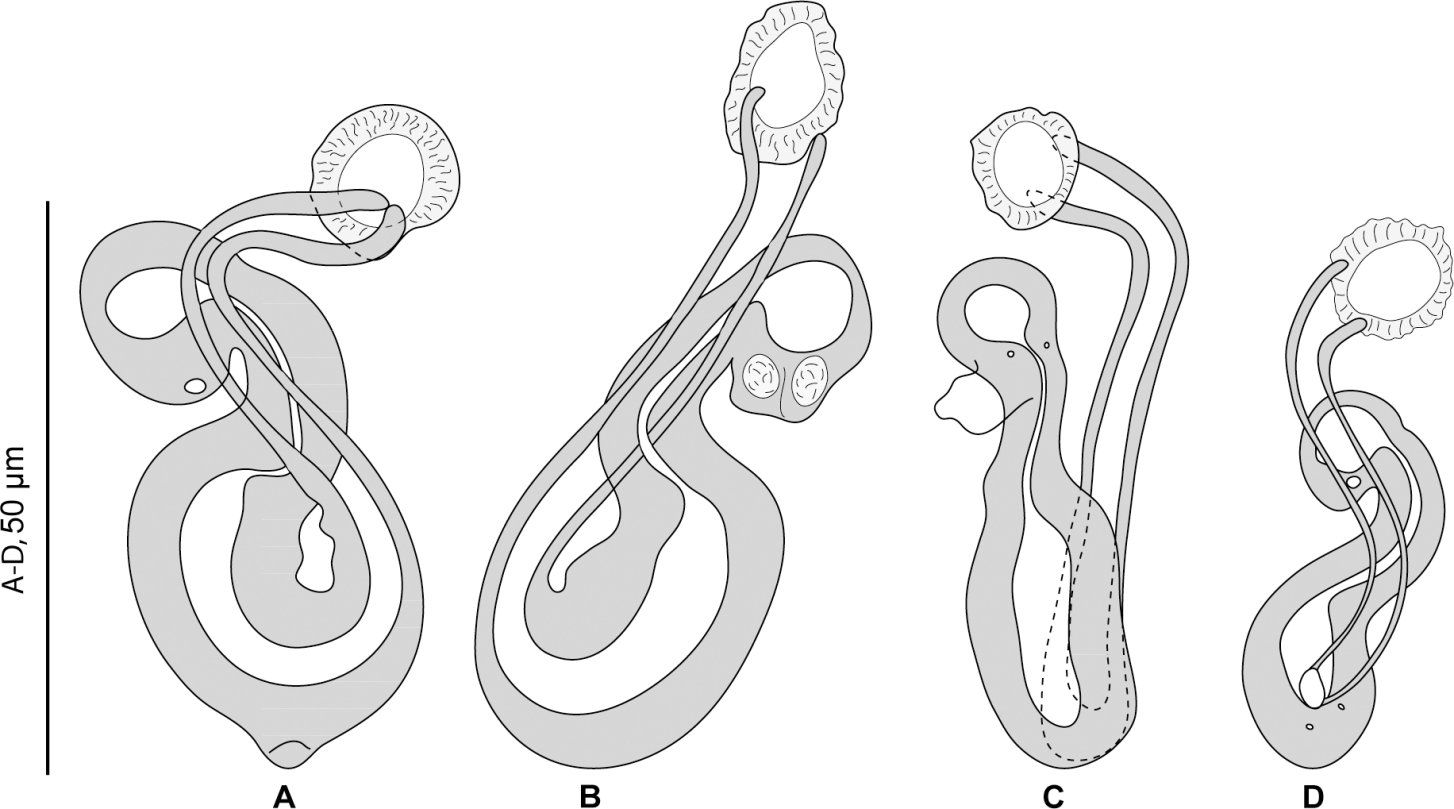
*Pseudorhabdosynochus hayet* n. sp. from *Mycteroperca rubra*, various shapes of sclerotised vagina according to specimens, orientation and preparation. A-D, different forms of vagina. A-C, Berlese. D, carmine.

*Sclerotised vagina* comprises sclerotised trumpet, primary canal, primary chamber, secondary canal and secondary chamber. Trumpet anterior, ring-shaped; primary canal with regular diameter and medium-thick wall, curved or S-shaped; connection between primary canal and primary chamber posterior; primary chamber pear-shaped or elongate, roughly parallel to primary canal but reversely oriented, its wall thick; secondary canal well visible, its lumen narrow; secondary chamber spherical, smaller than primary chamber; wall of secondary chamber thick; secondary chamber located just posterior to trumpet (Figs 6C and 7A–7D). Sclerotised vagina length h 45, c 46 (45–49, n = 4), p 43 (40–45, n = 3), b 61 (54–70, n = 5), diameter of primary chamber external h 10, c 10 (n = 4), p 9 (7–12, n = 3), b 15 (14–16, n = 5).

#### Differential diagnosis

*Pseudorhabdosynochus hayet* n. sp. can be distinguished from *P*. *oliveri* n. sp. by the shape of its primary canal (thin vs. wide in the latter), and from *P*. *beverleyburtonae* by the shape of its primary chamber (wide vs. thin in the latter). The morphology of the sclerotised vagina and other organs is very similar to that of *P*. *sosia*. The two species can, however, be distinguished by larger dimensions of the vagina in *P*. *hayet*. It is well known that measurements of sclerotised parts change with degree of flattening, i.e. the method for preparing slides [20]. Measurements between the two species are different when compared between specimens prepared with the same methods (Table 2): picrate (43 in *P*. *hayet* vs 35–36 in *P*. *sosia*) and Berlese (61 vs 44). The male copulatory organ is longer in *P*. *hayet*: picrate (89 vs 81–84 in *P*. *sosia*), and Berlese (150 vs 92). In addition, the hosts are different (*M*. *rubra* for *P*. *hayet* vs *M*. *costae* for *P*. *sosia*) (Table 1). The COI sequences are divergent (Table 4).

### The ‘beverleyburtonae group’

**Fig 8:**
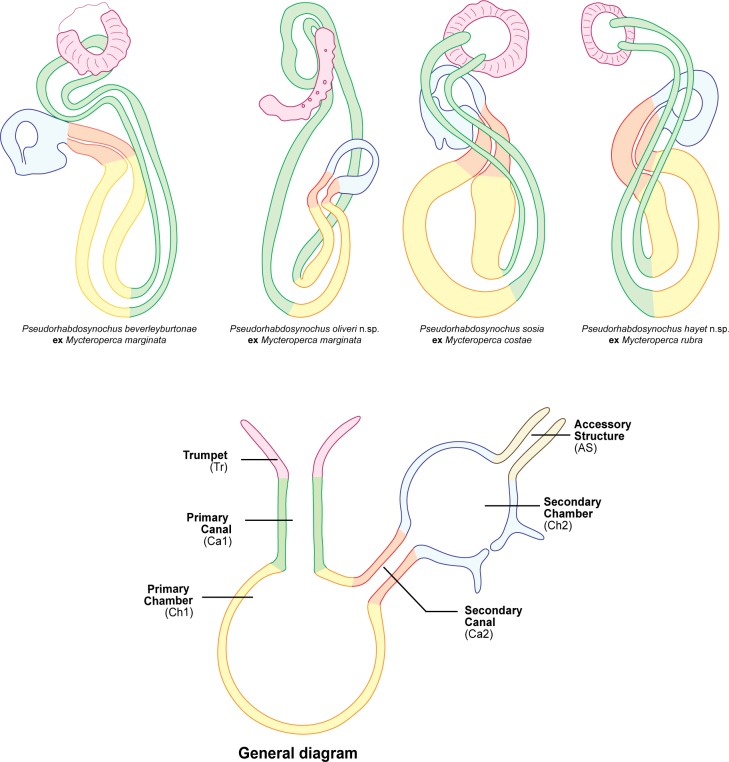
Homologies of the various parts of the sclerotised vaginae illustrated by coloured diagrams. The same colours are used in each diagram for the same parts, to show homologies between species. The nomenclature of vaginal parts and the general diagram are from Justine (2007) [1]. All vaginae drawn with same sizes–magnifications vary.

The four species assigned to *Pseudorhabdosynochus* described and/or redescribed herein, namely *P*. *beverleyburtonae* and *P*. *oliveri* n. sp. from *M*. *marginata*, *P*. *sosia* from *M*. *costae*, and *P*. *hayet* n. sp. from *M*. *rubra*, exhibit great similarity in the morphology of the sclerotised vagina, the main character on which *Pseudorhabdosynochus* species are distinguished [3, 4, 6, 32, 33]. Fig 8 shows a comparison of the sclerotised vagina in all four species; colours were used to show the various parts of the vaginae and the homologies between different species. In addition, all these four species are characterised by a similar structure of the squamodiscs, with two innermost rows as closed circles; this character was used as a primary characteristic to erect *Cycloplectanum* Oliver, 1968, which was later considered a junior synonym of *Pseudorhabdosynochus* [9] [28]. Squamodiscs with two innermost rows as closed circles are found in many *Pseudorhabdosynochus* species and even in species now placed in other genera such as *Echinoplectanum* Justine & Euzet, 2006 and thus cannot be used for defining a genus [34].

We propose, for these four species, a group which is referred to here as the ‘beverleyburtonae group’. The four species are parasitic on groupers of the genus *Mycteroperca* in the Mediterranean Sea and eastern Atlantic (with *P*. *beverleyburtonae* also found in the western Atlantic).

Three species of *Pseudorhabdosynochus* from groupers in the Mediterranean Sea share vaginal characters with species of the beverleyburtonae group but do not belong to it. These species are:

- *Pseudorhabdosynochus regius* Chaabane, Neifar & Justine, 2015, from *M*. *rubra* [6]. This species possesses the same general vaginal structure but it has a straight primary canal (vs curved or S-shaped in species of beverleyburtonae group), a less differentiated trumpet, and the primary and secondary chambers are grouped in a heavily sclerotised structure.

- *Pseudorhabdosynochus dolicocolpos* Neifar & Euzet, 2007, from *M*. *costae* [3]. This species has a well-defined trumpet but the primary canal is extremely long and thin, the primary chamber is very small and smaller than the secondary chamber.

- *Pseudorhabdosynochus sinediscus* Neifar & Euzet, 2007, from *M*. *costae* [3]. This species has a vaginal structure superficially resembling species of the beverleyburtonae group but there is no secondary chamber. In addition, it is distinguished by the lack of squamodiscs.

### Analysis of COI sequences of monogeneans

**Fig 9:**
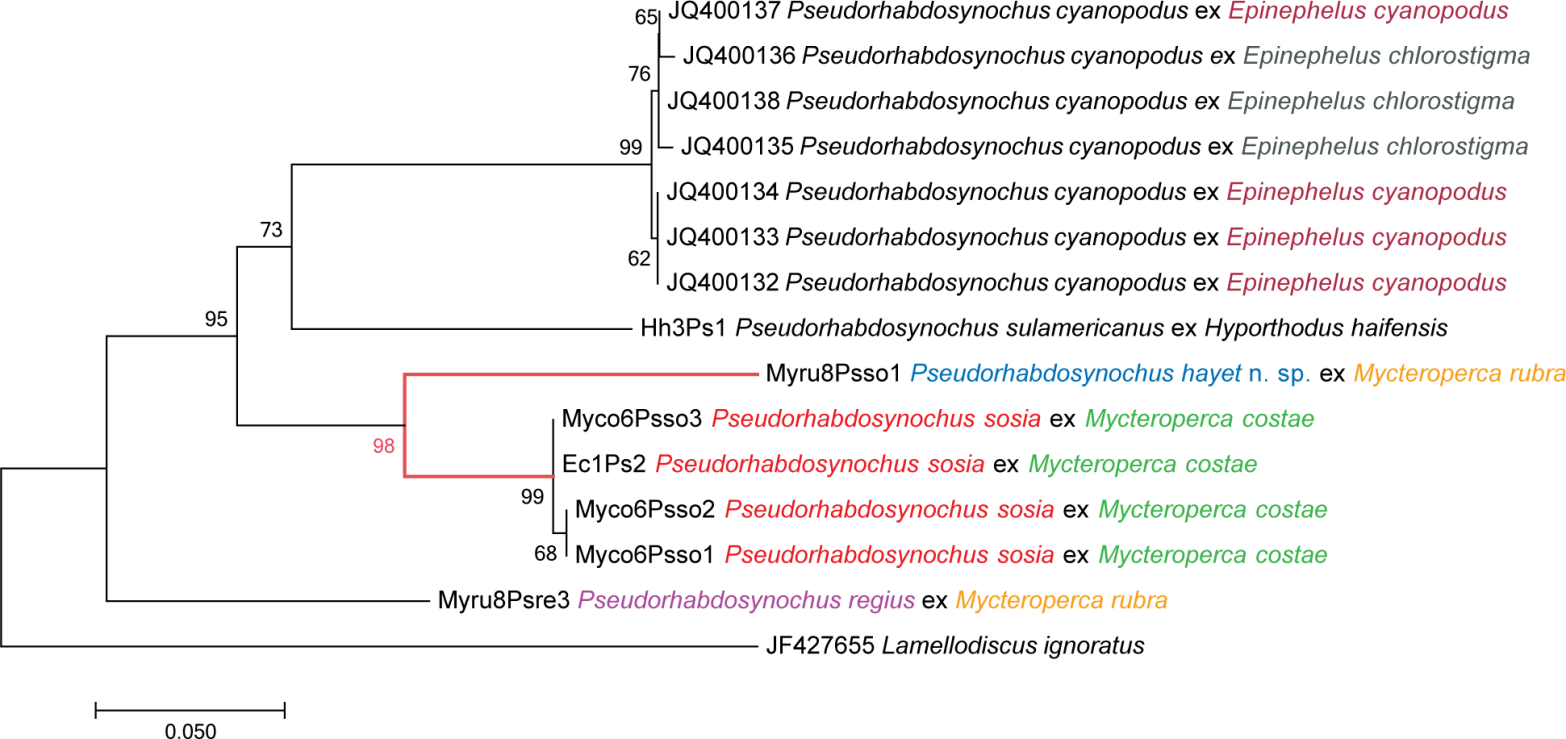
Evolutionary relationships of taxa. **The matrix includes new sequences of *Pseudorhabdosynochus* spp. and available sequences from GenBank.** The analysis, using the Neighbour-Joining method, involved 15 nucleotide sequences, and there were a total of 290 positions in the final dataset. Bootstrap test results (500 replicates) are shown next to the branches.

We obtained COI sequences of *P*. *hayet* n. sp. (1 specimen), *P*. *sosia* Neifar & Euzet, 2007 (4 specimens) and *P*. *regius* Chaabane et al., 2015 (1 specimen). The phylogenetic analysis was performed with these 6 new sequences and available sequences of *Pseudorhabdosynochus*, i.e. 1 sequence of *P*. *sulamericanus* Santos et al., 2000, 7 sequences of *P*. *cyanopodus* Sigura & Justine, 2008, and, as an outgroup, *Lamellodiscus ignoratus* Palombi, 1943, a member of the Diplectanidae family to which *Pseudorhabdosynochus* belongs. In the tree obtained by the NJ method (Fig 9), the two species *P*. *hayet* and *P*. *sosia* were sister-groups. The genetic distance between species of *Pseudorhabdosynochus* varied from 13 to 26%, and the distance between *P*. *hayet* and *P*. *sosia* was 13–14% (Table 4).

## Discussion

Gills of groupers (Epinephelidae) harbour numerous diplectanid monogeneans assigned to *Pseudorhabdosynochus* [1–5, 20, 29, 35–38]. This genus currently contains more than 80 valid species [39] which are generally specialists. Based on the great similarity in the shape of the sclerotised vagina, the primary character for *Pseudorhabdosynochus* species diagnosis, the ‘beverleyburtonae group’ is proposed herein to include two new and two previously described species on *Mycteroperca* spp. in the Mediterranean and Eastern Atlantic Ocean. Several groups of *Pseudorhabdosynochus* species have already been suggested such as those on *Epinephelus* spp. in the southern Pacific, namely the ‘*P*. *cupatus* group’ [35] (including *P*. *cupatus* (Young, 1969), *P*. *cyathus* Hinsinger & Justine 2006, *P*. *calathus* Hinsinger & Justine 2006, and three unnamed species) [20, 40], the ‘huitoe complex’ (including *P*. *huitoe* Justine, 2007, *P*. *manifestus* Justine & Sigura, 2007, and *P*. *crassus* Schoelinck & Justine, 2011) [1, 37, 41], and a small group that includes only *P*. *exoticus* Sigura & Justine, 2008 and *P*. *exoticoides* Justine & Henry, 2010 [38, 42]. Kritsky et al. 2015 also proposed three groups of *Pseudorhabdosynochus* species on *Epinephelus* spp., *Mycteroperca* spp., and *Hyporthodus* spp., respectively, in the western Atlantic Ocean [2]. These groups do not have formal systematic value but are useful for distinguishing species among the numerous valid *Pseudorhabdosynochus* species.

In this paper, we used mainly the morphology of the sclerotised vaginae to differentiate the diplectanid species. However, we added molecular information on two species for which the differentiation was mainly based on differences of measurements, but not on differences of morphology, namely *P*. *sosia* and the new species *P*. *hayet*. Using multiple and complementary sources of data, i.e. ‘integrative taxonomy’ [43], is important for better species characterisation and delimitation. In the case of *P*. *sosia* and *P*. *hayet*, the fact that the hosts are different is additional information advocating different parasite species, but is certainly not sufficient because it has been demonstrated that, in some cases, the same *Pseudorhabdosynochus* species can parasitize different host fish [44]; in that particular case, the genetic differences between specimens of *P*. *cyanopodus* Sigura & Justine, 2008 from two hosts were very low (0–1.5%). The genetic distance between *P*. *sosia* and *P*. *hayet* was 13–14%, thus lower than the differences observed between other *Pseudorhabdosynochus* species in our dataset (19–26%), but much higher than the differences between specimens of *P*. *cyanopodus* found on two different hosts [44]. The literature on monogenean COI sequences and their interspecific distances is scarce. Vanhove et al. (2013) have emphasized the difficulties encountered in using COI for the differentiation of species in various groups of flatworms, including monogeneans; however, they cite a case in which two valid diplectanid species were divergent by only 3.2% [45]. In other flatworms (geoplanid triclads), such as *Microplana* spp., intraspecific variation was up to 4%, and interspecific variation was 19% [46], and a difference of 4.8% between *Platydemus manokwari* haplotypes was considered intraspecific [47]. New species are hypotheses that should be tested [48]. We conclude that the 13–14% difference of COI sequences between *P*. *sosia* and *P*. *hayet* is indicative of two different species, in addition to morphological differences and different hosts, and that *P*. *hayet* should be erected as a new species.

A list of 10 grouper-diplectanid species in the Mediterranean Sea has been provided (see Table 1 in [6]), and another species, *P*. *sulamericanus*, was recently added [6, 7]. The two new species of *Pseudorhabdosynochus* described here bring the total of species known in the Mediterranean to thirteen; however, the biodiversity of diplectanids on Mediterranean groupers is not yet fully documented.
